# NEURONAL FGF-21 SIGNALING: A SENSOR OF DIETARY PROTEIN

**DOI:** 10.1093/geroni/igz038.2677

**Published:** 2019-11-08

**Authors:** Cristal Hill, Christopher Morrison

**Affiliations:** Pennington Biomedical Research Center, Baton Rouge, Louisiana, United States

## Abstract

Our data demonstrates that dietary protein restriction increases energy expenditure and improves glucose homeostasis, and that this effect is largely mediated by the metabolic hormone fibroblast growth factor 21(FGF21). Considering that the central nervous system (CNS) is acknowledged as a major regulator of both energy and glucose homeostasis, we have extended our studies to identify the tissue site mediating these FGF21-dependent effects via dietary protein restriction. In this study, mice with dysfunctional FGF21-signaling in either the CNS or adipose tissue were fed a control or low protein (LP)-diet to assess changes in body weight and metabolic endpoints. Our data show that LP diet increased energy expenditure and reduced body weight in control littermates, but these effects were lost in mice bearing CNS-specific deletion of Klb. These data highlight a liver to brain FGF21-signal as the first known neuroendocrine mechanism to explain the coordinated metabolic changes induced by dietary protein restriction.

